# Effects of Pre- and Post-Processing on Pin-Bearing Strength of 3D-Printed Composite Specimens with Circular Notches

**DOI:** 10.3390/polym17192609

**Published:** 2025-09-26

**Authors:** Yong-Hun Yu, Do-Hyeon Kim, Kang Rae Cho, Hyoung-Seock Seo

**Affiliations:** 1 Department of Autonomous Vehicle System Engineering, Chungnam National University, Daejeon 34134, Republic of Korea; usefulhun@o.cnu.ac.kr; 2 Hyundai Heavy Industries, Ulsan 44032, Republic of Korea; kdh6479@hd.com; 3 Department of Advanced Materials Chemistry, Cheongju University, Cheongju 28503, Republic of Korea; chokangrae@gmail.com

**Keywords:** 3D-printing, continuous fiber-reinforced polymer (CFRP), bearing test, pre- and post-processing, fiber orientation, failure mode

## Abstract

To apply 3D printing-based continuous fiber composites in shipbuilding and marine applications, the pin-bearing fastening method with notch holes can be considered as an effective method. In this study, pin-bearing strength tests were performed on a 3D-printed composite consisting of carbon fiber and Onyx to evaluate the effect of hole notches fabricated through pre- and post-processing. The experimental results showed the difference in the mechanical fastening strength of the specimens, depending on the method used to fabricate the hole notch. As the width-to-diameter ratio (W/D) decreased, ultimate bearing strength, strain, and toughness decreased. The post-treated specimens exhibited higher initial stiffness than the pre-treated specimens, and their bearing stress was up to 23% higher at smaller hole diameters (≤6 mm). In particular, for specimens with 0° fiber orientation, the post-processed specimens showed markedly higher toughness than the pre-processed ones, with increases at 5 mm and 6 mm hole diameters, respectively, thereby demonstrating superior performance in both strength and energy absorption. The damage modes of the circular notches were also found to depend on the pre- and post-processing conditions. These results suggest that fiber orientation, W/D ratio, and processing method should be considered when designing mechanical fasteners for 3D-printed composites in marine structures.

## 1. Introduction

The International Maritime Organization (IMO) has established a strategy to reduce total greenhouse gas emissions from the shipping sector by at least 50% by 2050 [1]. In compliance with environmental regulations, lightweighting of ship structures has emerged as an important task [2]. Improving fuel unit efficiency through lightweighting means reducing greenhouse gas emissions while improving ship performance through efficient fuel use [3,4,5]. To achieve these goals, 3D printing composite materials and 3D printing manufacturing technology are good candidates to provide high strength to stiffness ratios, which can reduce weight when applied to various marine structures such as hulls, rudders, and pipe [6,7,8]. The use of fiber-reinforced composites through 3D printing manufacturing technology has proven its excellence in the aerospace, defense, and unmanned vehicle industries where high-performance weight reduction is essential [9,10,11]. Three-dimensional printing technology also offers diverse advantages of design flexibility, complexity, and rapid prototyping [12]. Invernizzi et al. [13] used CFRP to produce airfoils, blades, and propellers with complex CAD models. Continuous fiber-reinforced composites can be fabricated using Fused Deposition Modeling (FDM) technology in 3D printing. The FDM of 3D printing technology can be expected to have weight reduction and manufacturing cost reduction effects by controlling variables such as infill pattern, fiber orientation direction, and topology optimization inside the structure [14,15,16].

To apply 3D printed composites to ships and marine structures, it is necessary to consider not only durability in the marine environment but also the method of connecting the structures [17]. Since welding is not an appropriate method for composite materials like metal structures, adhesives and mechanical fastening are used [18]. Due to the incompatibility of conventional welding methods used for metals with composite materials, mechanical fastening is typically utilized [19]. It has the advantage of excellent durability against assembly, disassembly, part replacement, repair, and environmental influences [20,21,22,23]. On the other hand, when using mechanical fastening, there are problems such as stress concentration due to the hole notch and reduced load-bearing capacity due to damage to fiber continuity during drilling [24,25,26]. Extensive research has been conducted on geometric parameters, such as hole diameter, due to their significant influence on load-bearing capacity and failure modes.

Othman et al. [27] conducted experiments to investigate the effects of geometric parameters on GFRP composite specimens. It was confirmed that geometric parameters such as hole diameter (D), specimen width (W), and the edge distance (E) from the center of the hole to the specimen’s end significantly influence the failure mode. Specifically, specimens exhibited tensile failure at low W/D ratios and shear failure at low E/D ratios. These findings indicate that a stable bearing failure mode can be achieved when certain critical thresholds of geometric parameters are exceeded. D.U. Kim et al. [28] studied the bearing strength of specimens according to geometric effects using CFRP and GFRP specimens. The experimental results confirmed that CFRP had 30% better strength than GFRP regardless of the hole diameter. In addition, it was confirmed that the maximum bearing load increased as W/D decreased. Calabrese et al. [29] investigated the failure shape that changes according to geometric parameters and developed a failure map. It was observed that the bearing stress exhibits a decreasing trend with larger hole diameters, whereas it increases linearly with greater edge distances from the hole center to the specimen boundary, before converging beyond a critical threshold. J.S. Oh et al. [30] investigated the effect of fiber orientation on the bearing strength of 3D-printed composite laminates. They observed that specimens with fibers aligned at 0° exhibited significantly higher bearing strength than those with ±45° or 90° orientations, and that the sensitivity of bearing strength to hole diameter strongly depended on fiber alignment. Xu et al. [31] reviewed the drilling of CFRP materials, focusing on fundamental mechanisms and damage formation. They highlighted that drilling parameters such as cutting speed, feed rate, and tool geometry directly influence delamination, fiber pull-out, and uncut fibers, which in turn deteriorate the load-bearing capacity of mechanically fastened joints.

Many studies have been conducted on the geometrical effects on the mechanical fastening of specimens manufactured by traditional lamination and processing methods of composite materials. However, there is little research on the effects of pre-processing methods that form the circular notch in advance in the 3D printing manufacturing process and post-processing methods that form the circular notch as a post-processing process after lamination on the pin fastening strength.

In this study, the effects of the pre-processing and post-processing methods of 3D printing-based CFRP specimens on the pin fastening strength are quantitatively investigated, including the circular diameter and fiber orientation variables. In addition, the effects of each variable on the internal failure mode are quantitatively investigated through the fracture analysis of the fastening part.

## 2. Materials and Methods

### 2.1. Material and Fabrication Method

There are several 3D printing methods, including Fused Deposition Modeling (FDM), Digital Light Processing (DLP), Selective Laser Sintering (SLS), and Stereolithography (SLA). Among them, FDM is the most widely used due to its simplicity and low cost, as it deposits successive layers of thermoplastic filament through a heated nozzle.

**Figure 1:**
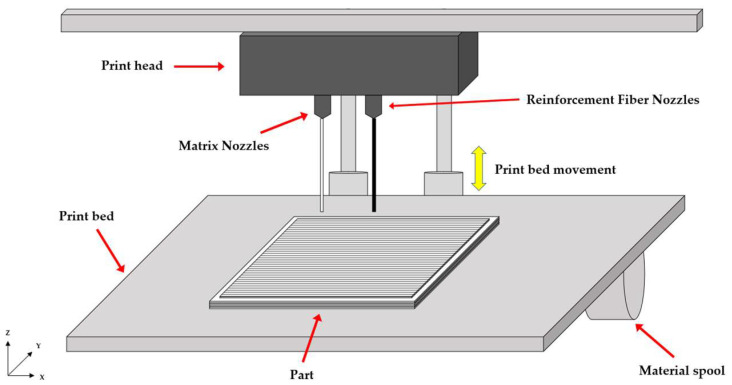
Schematic of CFF 3D print method.

Markforged, a company specializing in advanced additive manufacturing, utilizes a technique called Continuous Filament Fabrication (CFF), an extension of FDM. In this process, a high temperature dual nozzle print head melts and extrudes both the base thermoplastic and continuous fiber filaments through the matrix and reinforcement nozzles, respectively, to fabricate parts layer by layer, as illustrated in Figure 1.

The CFF method enables continuous fiber impregnation, resulting in printed components with enhanced mechanical performance.

In this study, composite specimens were printed using the Markforged X7™ system (Markforged, Waltham, MA, USA), which employs the CFF technique. The specimens were made from continuous carbon fiber and Onyx, whose material properties are listed in Table 1.

### 2.2. Three-Dimensional Printed Composite Material Specimen Fabrication

**Figure 2:**
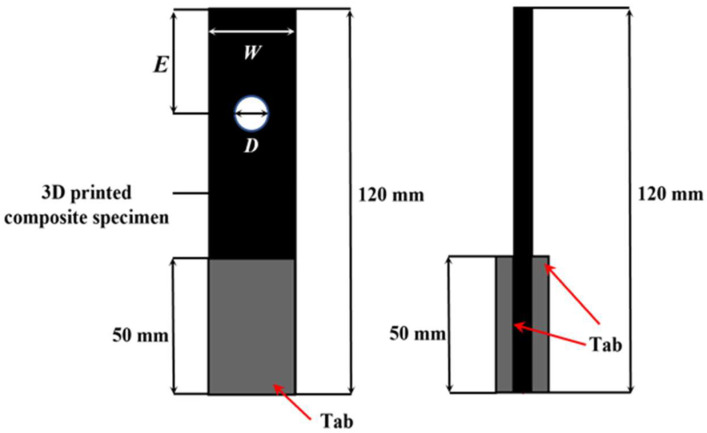
Schematic of 3D printed bearing specimen.

The geometry and dimensions of the specimens used to evaluate the pin-bearing strength are presented in Figure 2 and Table 2. Each specimen had a fixed total length of 120 mm, while the circular hole diameters (D) were varied across 5, 6, 8, and 10 mm to assess the influence of geometric changes. The specimen width (W) and the distance from the hole center to the specimen edge (E) were kept constant. As a result, the width to diameter ratio (W/D) and edge distance to diameter ratio (E/D) varied with hole size, as summarized in Table 2.

Each layer of carbon fiber and Onyx filament was printed with a thickness of 0.125 mm. The upper and lower sections of the specimen consisted of four layers of Onyx each, yielding a thickness of 0.5 mm per section. The central section was composed of 16 layers of continuous carbon fiber, resulting in a thickness of 2.0 mm. Consequently, the total thickness (t) of the specimen was 3.0 mm, comprising 24 laminated layers fabricated using the fused deposition modeling (FDM) process.

**Figure 3:**
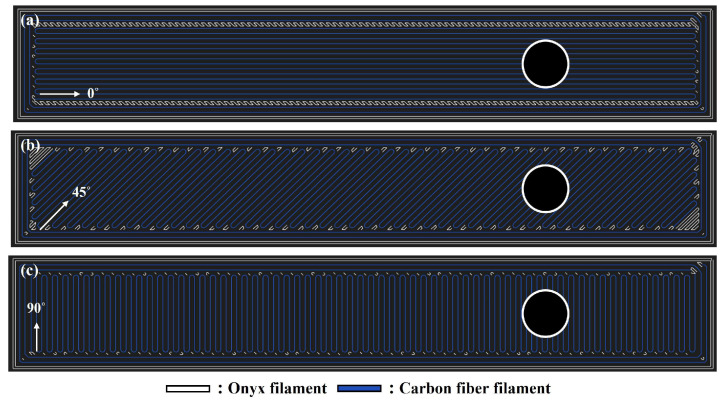
Markforged S/W Eiger software (Markforged Inc, available online: https://www.markforged.com/eiger accessed on 12 March 2025) image: Printing direction of Core laminate (**a**) 0°; (**b**) 45°, (**c**) 90°.

To evaluate the influence of printing direction and geometric factors on mechanical fastening strength and fracture behavior, the central laminate was printed in three orientations: 0°, ±45°, and 90°, as illustrated in Figure 3. Detailed laminate configurations are summarized in Table 3.

Unlike conventional manufacturing methods, 3D printing offers the advantage of eliminating the need for molds and enabling the direct fabrication of features such as holes without post-processing. To examine the effects of hole fabrication methods, two types of specimens were prepared. In Type 1 specimens, holes were printed directly during the 3D printing process. In Type 2 specimens, a rectangular plate with identical dimensions (L, W, t) was printed, and the holes were subsequently introduced via post-processing.

**Figure 4:**
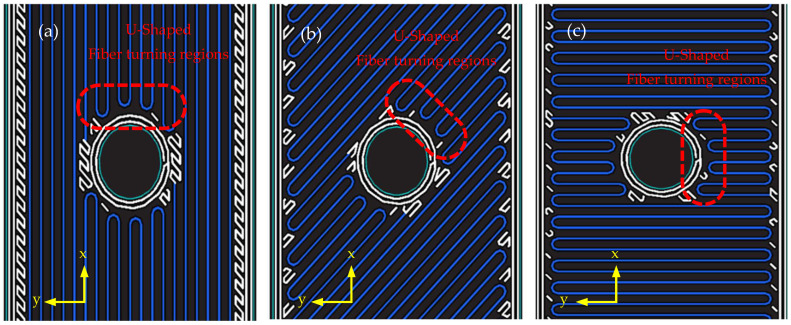
Fiber rotation section of Type 1: (**a**) 0°; (**b**) ±45°. (**c**) 90°.

In the case of the pre-processing specimens, holes were generated directly during printing. Figure 4 illustrates the filament paths around the printed holes. At the hole boundary, the Onyx material formed a circular contour, while the carbon fiber filaments followed the assigned layer orientation. Since the filaments could not pass through the hole, they curved back in short rounded paths near the boundary, creating local turning regions. The characteristics of these regions varied with fiber orientation and hole size.

For the post-processing specimens, circular holes were drilled manually at low speed to minimize thermal damage and delamination. A standard carbide twist drill (two cutting edges, 118° tip angle, 30° helix angle) was used, and drilling was performed slowly under manual feed (cutting speed < 200 rpm, feed rate < 0.1 mm/rev). These conditions were chosen to avoid excessive heat generation and resin melting, which can occur under high-speed machining of composite materials.

Although post-processing allows for more precise hole dimensions, it can also introduce defects that may compromise mechanical fastening strength [31]. To evaluate the effects of these fabrication methods on strength and fracture behavior, pin-bearing tests were conducted on both specimen types.

### 2.3. Double-Lab Fixture and Pin

The double-lap fixture and pin used in the bearing tests were fabricated from stainless steel. The pin was 50 mm in length and was fabricated with diameters of 5, 6, 8, and 10 mm to match the corresponding hole sizes.

**Figure 5:**
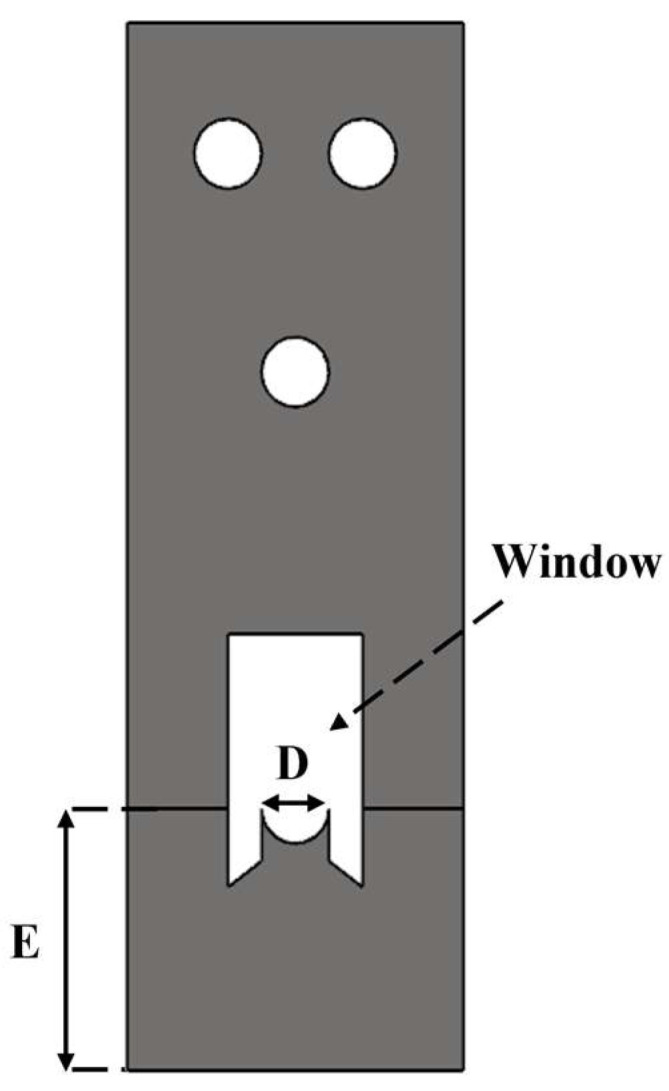
Double-lab fixture to investigate the bearing damage of specimen.

To monitor crack initiation and propagation during testing, a viewing window was incorporated into the fixture, as shown in Figure 5. This window allowed real-time observation of crack growth throughout the test. A protrusion within the window secured the pin in place, and four fixtures were prepared to correspond to each pin diameter.

### 2.4. Test Setup

**Figure 6:**
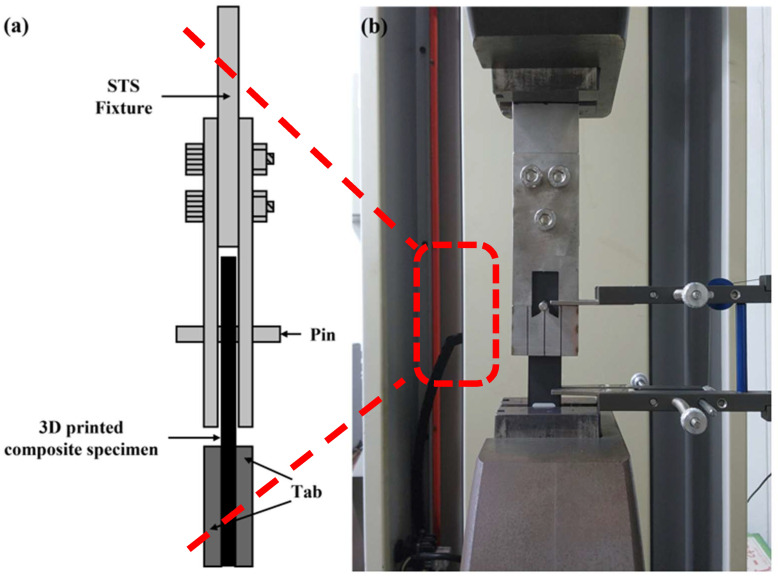
Experimental set-up: (**a**) Schematic of the fastened specimen; (**b**) Specimen installed in UTM.

The bearing test was conducted using a UTM (Universal Testing Machine, Daekyung Tech, Incheon, Republic of Korea), in accordance with the ASTM D5961 standard [33]. The loading rate was set to 2 mm/min, and the test was terminated at 70% of the maximum load. The specimen was secured in the double-lap fixture using the pin, and the assembly was mounted in the testing machine, as illustrated in Figure 6.

### 2.5. Stress Concentration and Distribution

The ultimate bearing stress and bearing strain according to the mechanical fastening of composite materials were calculated according to ASTM D5961 [33].

(1) $$\sigma^{bru}=\frac{P}{\left(k\times D\times h\right)}$$

Equation (1) is the equation for the ultimate bearing stress. Here, $\sigma^{bru}$ is the bearing stress (MPa), P is the load (N), *D* is the hole diameter (mm), *h* is the specimen thickness (mm), and *k* is the load factor per hole. For single fastener and pin experiments, *k* is 1.0, and for double fasteners, 2.0.

(2) $$\varepsilon^{br}=\frac{\delta }{\left(K\times D\right)}$$

Equation (2) is the equation for bearing strain. Here, $\varepsilon^{br}$ is the bearing strain, δ is the displacement measured by an extensometer (mm), *D* is the hole diameter (mm), and *K* is a constant, equaling 2.0 for a single shear test and 1.0 for a double shear test.

Toughness, a measure of a material’s ability to absorb energy prior to fracture, was determined by calculating the area under the stress–strain curve [30]. This parameter is critical in evaluating the structural durability and damage tolerance of composite joints.

## 3. Results and Discussion

### 3.1. Mechanical Fastening Strength Evaluation

In total, 24 different test cases were considered, covering all combinations of fiber orientation, hole diameter, and processing method. For each case, three specimens were fabricated and tested independently.

Stress and strain were calculated from the load–displacement data using Equations (1) and (2), and toughness was determined as the area under the stress–strain curve. The area was obtained by numerically integrating stress with respect to strain up to the failure point, which represents the absorbed energy per unit volume (J/m^3^). Structure stiffness was evaluated from the initial linear portion of the load–displacement curve. The slope of the best-fit line within the selected linear region was taken as the stiffness, expressed in N/mm.

Table 4 summarizes the average values of ultimate bearing stress, bearing strain, and damage energy for both pre-processed and post-processed specimens across all test conditions.

**Figure 7:**
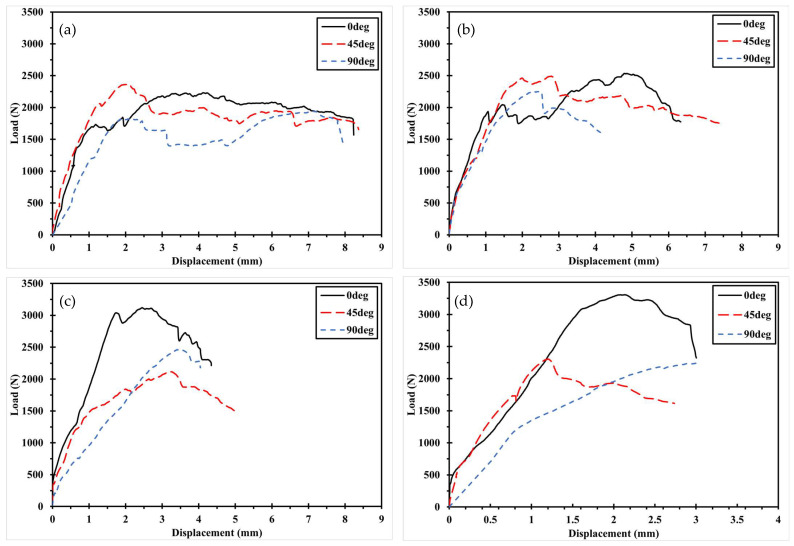
Load–displacement curve according to printing direction of Type 1: (**a**) D5 specimen; (**b**) D6 specimen. (**c**) D8 specimen. (**d**) D10 specimen.

**Figure 8:**
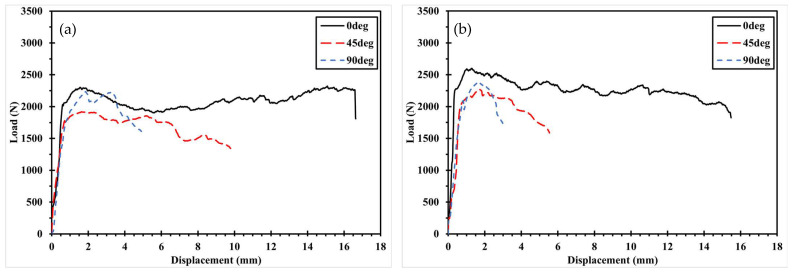

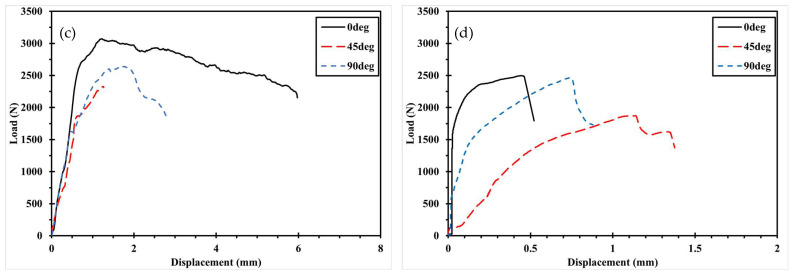
Load–displacement curve according to printing direction of Type 2: (**a**) D5 specimen; (**b**) D6 specimen. (**c**) D8 specimen. (**d**) D10 specimen.

The mechanical fastening behavior of the 3D-printed composite specimens, classified by pre- and post-processing methods, is illustrated in Figure 7 and Figure 8 with respect to printing direction. Figure 7 shows the load–displacement curves of Type 1 specimens, where the circular holes were directly printed. Specimens printed at 0° exhibited fracture loads that were 2.5% to 69.6% higher than those printed at ±45° or 90°, under the same W/D ratio. Similarly, Figure 8 presents the results for Type 2 specimens, in which holes were introduced through post-processing. In this case, the 0° specimens showed fracture loads 2.7% to 35.5% higher than those with other fiber orientations. This trend is attributed to the alignment of fibers in the loading direction, which provides greater resistance to the applied force.

**Figure 9:**
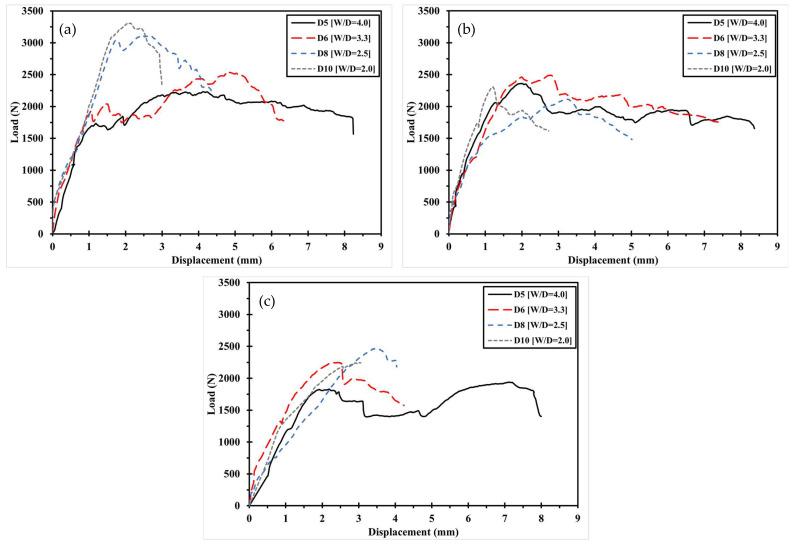
Load–Displacement curve with increasing hole diameter in the same fiber orientation of Type 1: (**a**) 0°; (**b**) ±45°. (**c**) 90°.

**Figure 10:**
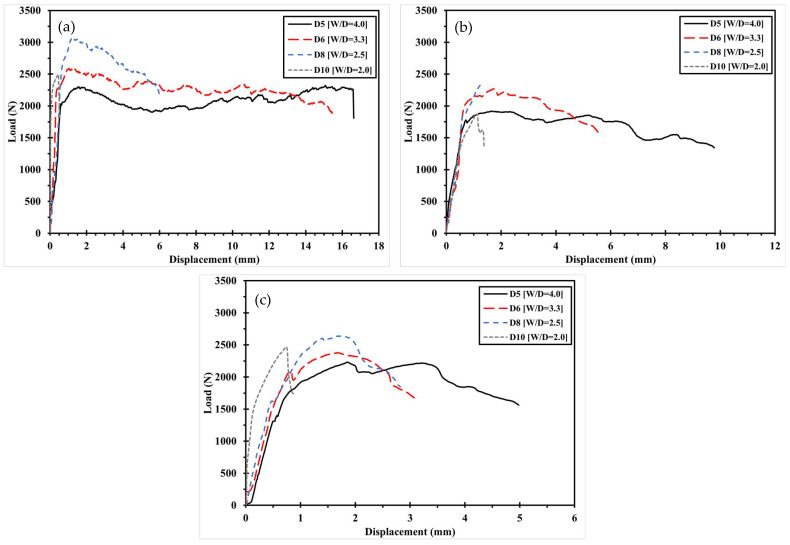
Load–Displacement curve with increasing hole diameter in the same fiber orientation of Type 2: (**a**) 0°; (**b**) ±45°. (**c**) 90°.

Figure 9 and Figure 10 illustrate the variation in mechanical fastening strength with increasing hole diameter for specimens with the same fiber orientation. In general, the maximum bearing load increases with hole diameter due to the enlarged contact area between the pin and the specimen, which reduces the stress per unit area and thereby mitigates stress concentration. However, in Figure 9b, a deviation from this trend is observed the maximum bearing load decreases when the hole diameter increases from 6 mm to 8 mm, and again from 8 mm to 10 mm. This reduction is attributed to a shift in the fracture mode, which will be discussed further in Section 3.3.

**Figure 11:**
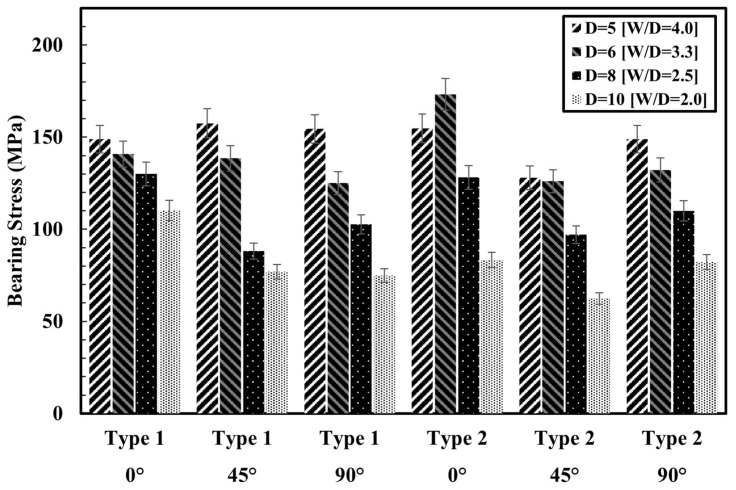
Ultimate bearing stress of pre- and post-processed specimens.

Figure 11 presents the ultimate bearing stress of pre- and post-processed specimens with identical fiber orientations. Overall, bearing stress tends to decrease as the W/D ratio decreases, despite an increase in the applied load. This is because the increase in hole diameter leads to a greater reduction in stress per unit area, primarily due to reduced contact stiffness and elevated stress concentrations [28,30]. For specimens with 0° fiber orientation, the post-processed group exhibited 4% and 23% higher bearing stress at hole diameters of 5 mm and 6 mm, respectively, compared to the pre-processed group. However, this advantage reversed at larger hole diameters (8 mm and 10 mm), likely due to thermal effects and microstructural damage introduced during drilling. A similar trend was observed for bearing strain. The post-processed specimens showed strain values that were 130.6% and 206.7% higher at 5 mm and 6 mm, indicating improved energy absorption capacity. At 10 mm, however, strain dropped by 83.9% compared to the pre-processed specimen, suggesting significant interface degradation caused by accumulated processing damage.

**Figure 12:**
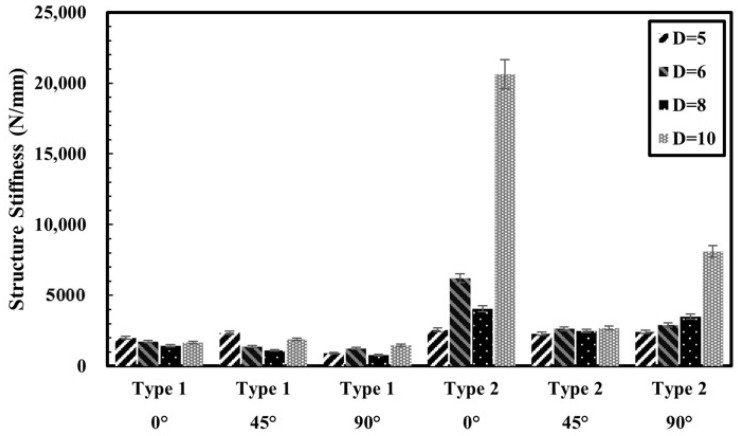
Structure stiffness of specimens.

As shown in Figure 12, post-processed specimens exhibited increased initial stiffness across most hole diameters. For 0°-oriented specimens, the initial slope in the linear region increased by 183% to 1145.5% compared to the pre-processed group, particularly when the hole diameter was 6 mm or larger. This enhancement may be associated with local compression of the matrix and fibers during drilling, which increases resistance at the early stage of loading. In the case of 90°-oriented specimens, where fiber contribution is minimal and the matrix dominates the mechanical response, post-processed specimens still showed stiffness increases ranging from 131.9% to 451.0%. These results suggest that post-processing may help relieve residual stresses and heal micro defects within the printed structure through localized remelting under heat and pressure. Overall, the data indicate that post-processing can be effective in enhancing the initial stiffness of 3D-printed composites, regardless of fiber orientation.

### 3.2. Toughness

**Figure 13:**
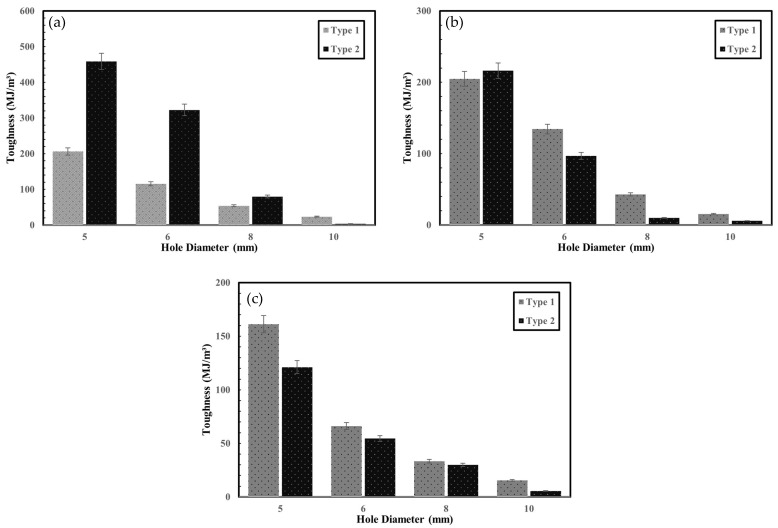
Toughness of specimens: (**a**) 0°; (**b**) ±45°. (**c**) 90°.

Toughness represents the energy absorbed by a material before fracture and serves as an indicator of the fracture resistance of composite joints. In marine environments, where structures are subjected to wave loads, fatigue, and impact, the toughness of joints is a critical factor in design. As shown in Table 4 and Figure 13, toughness consistently decreased with decreasing W/D ratio across all printing directions, reflecting the influence of geometry on joint performance.

For specimens with 0° fiber orientation, post-processed (Type 2) specimens exhibited 122% and 179% higher toughness at 5 mm and 6 mm hole diameters, respectively, compared to pre-processed (Type 1) specimens. However, at 10 mm, the toughness of post-processed specimens was 84% lower.

In the case of the ±45° orientation, pre-processed specimens generally showed higher toughness than post-processed ones, except at the 5 mm hole diameter, where the post-processed specimens exhibited greater values. At 90°, the pre-processed specimens consistently exhibited greater toughness across all hole diameters. This trend can be explained by the role of the Onyx around the hole. In the pre-processing method, Onyx forms a wall layer surrounding the hole, which helps resist crack propagation and absorb energy, particularly when the fibers are not aligned with the loading direction [30]. By contrast, in the post-processing method, the holes are drilled after printing, leaving no Onyx reinforcement around the hole edge. As a result, the fibers oriented at ±45° or 90° are less effective in sustaining the applied load, leading to lower toughness compared with pre-processed specimens.

In contrast, at 0° orientation, the post-processed specimens exhibited higher toughness than the pre-processed ones. This is because in pre-processing, the fibers curve around the hole and are interrupted by the Onyx contour, reducing their load-bearing efficiency. In post-processing, however, the fibers remain continuous across the hole region, enabling more effective load transfer along the loading direction.

Overall, these findings indicate that the relative performance of pre- and post-processing depends not only on W/D ratio but also on fiber orientation and the presence of the Onyx around the hole. When fibers are aligned with the load (0°), continuity of reinforcement in post-processing enhances toughness, while at ±45° and 90°, the supporting role of the Onyx layer in pre-processing provides superior resistance.

### 3.3. Failure Mode

**Figure 14:**
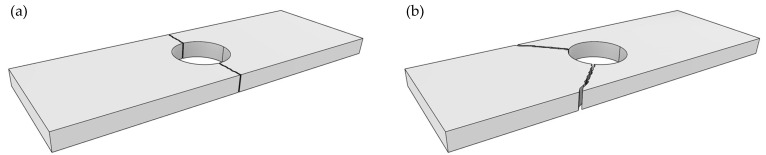

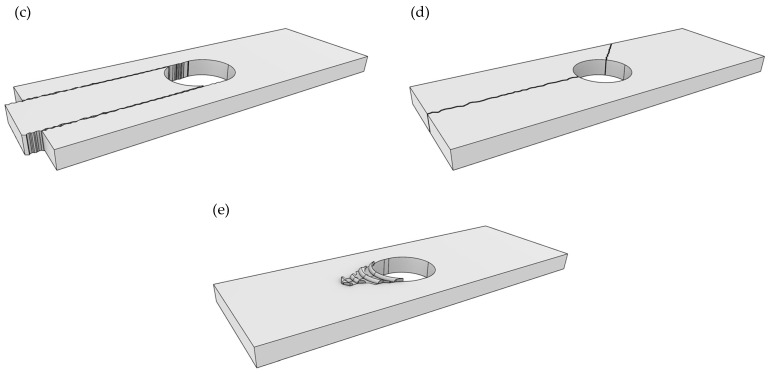
Bearing test failure mode: (**a**) Net-tension failure mode; (**b**) Tear-out failure mode. (**c**) Shear-out failure mode. (**d**) Cleavage failure mode. (**e**) Bearing failure mode.

When composite materials are connected using mechanical fasteners, several distinct failure modes may occur, each affecting the maximum bearing load the structure can sustain. According to the ASTM D5961 standard [33], these failure modes are illustrated in Figure 14.

Net-tension failure occurs when the tensile strength is insufficient, either due to a lack of fiber reinforcement in the loading direction or inadequate specimen width adjacent to the hole. Tear-out failure typically arises when fibers are oriented at ±45°, and tensile loads cause the material between the hole and the specimen edge to tear out. This mode is closely related to net-tension failure but occurs at different fiber orientations. Shear-out failure occurs when the shear strength of the material is lower than the applied load, often due to inadequate fiber reinforcement at angles that resist shear. Cleavage failure involves crack propagation in the longitudinal direction under tensile loading. This results in bending moments across the cross-section and is often accompanied by a simultaneous net-tension failure on the opposite side of the crack. Bearing failure occurs when the material has sufficient tensile and shear strength, but localized stress concentrations at the hole exceed the material’s bearing capacity, leading to surface indentation or localized crushing.

**Figure 15:**
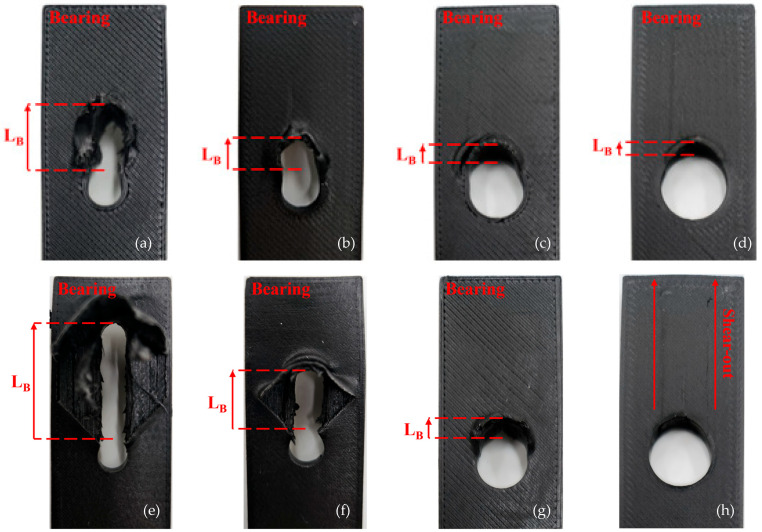
Failure mode of 3D printing bearing specimen (0°) of Type 1: (**a**) D5 specimen; (**b**) D6 specimen. (**c**) D8 specimen. (**d**) D10 specimen. Type 2: (**e**) D5 specimen; (**f**) D6 specimen. (**g**) D8 specimen. (**h**) D10 specimen.

The failure modes observed in the 3D-printed composite specimens after the bearing tests are summarized in Table 5. The failure modes in Table 5 were investigated from three independent tests for each condition, and representative images for the 0° fiber orientation are presented in Figure 15. These images illustrate how the failure mode changes with increasing hole diameter, and the observed patterns are consistent with the typical damage modes reported for conventional CFRP composites [34].

For Type 1 specimens printed at 0°, all cases exhibited bearing failure. However, as shown in Figure 15, the damage length (LB) decreased with increasing hole diameter. This observation is consistent with previous studies, which reported that increased hole size and reduced W/D ratio intensify stress concentration and influence damage propagation in composite joints [24,27,29]. In Type 1 specimens with ±45° orientation, failure modes could be categorized into two groups bearing failure occurred at 5 mm and 6 mm hole diameters, while tear-out or combined modes appeared at 8 mm and 10 mm. This indicates that the range of 2.5 ≤ W/D ≤ 3.3 represents a critical transition zone for failure mode change [28]. Similarly, in Type 1—90° and all Type 2 specimens, the failure mode transition was observed in the range 2.0 ≤ W/D ≤ 2.5. This explains why the maximum bearing load does not consistently increase with hole diameter; beyond a certain geometric threshold, the failure mode shifts to a less favorable one [28]. Importantly, none of the specimens experienced complete structural separation. This is attributed to the presence of the wall layers at both ends of the specimens, which served as physical constraints and prevented total detachment during loading.

## 4. Conclusions

This study investigated the mechanical fastening behavior of 3D-printed continuous fiber composites, focusing on the effects of pre- and post-processing methods. A total of 24 specimens with varying fiber orientations and hole diameters were fabricated and tested. The analysis included measurements of ultimate bearing load, bearing strain, initial stiffness, toughness, and failure modes.

- In general, for specimens with the same fiber orientation, a decrease in the W/D ratio led to reductions in ultimate bearing strength, strain, and toughness. This trend was particularly pronounced in post-processed specimens with 0° fiber orientation, due to cumulative damage introduced during drilling as the hole diameter increased.
- Across all configurations, post-processed specimens exhibited higher initial stiffness than pre-processed ones. The improvement was especially notable at 90° fiber orientation, possibly related to localized changes in the matrix during post-processing.
- Type 1 specimens with 0° fiber orientation consistently exhibited bearing failure, with the damage length decreasing as the hole diameter increased. In contrast, post-processed specimens showed longer damage lengths and transitioned to shear failure at larger diameters (10 mm).
- For specimens with ±45° orientation, failure modes shifted depending on the hole size, and the range 2.5 ≤ W/D ≤ 3.3 was identified as a critical region for mode transition. No complete delamination was observed, which is attributed to the presence of wall layers that helped restrain total detachment during loading.

These findings indicate that post-processing can enhance strength, stiffness, and toughness at smaller hole diameters (≤6 mm), but its benefits appear to diminish with increasing hole size, likely due to accumulated damage. In addition, the effect of the processing method depends strongly on fiber orientation. When the fibers are aligned with the loading direction (0°), post-processed specimens exhibit superior toughness and overall performance. At ±45° and 90°, pre-processed specimens generally show higher toughness than the post-processed ones. Therefore, when applying 3D-printed composites to ship and marine structures, careful consideration of the W/D ratio, fiber orientation, and post-processing strategy is essential. Incorporating end wall layers is also recommended to improve structural integrity under mechanical fastening. In practical design for shipbuilding and marine applications, it is important to account for the distinct advantages of both pre- and post-processing approaches. Post-processing can improve toughness and stiffness in smaller fastener holes, whereas pre-processing ensures more stable performance in larger holes. Thus, designers should integrate these complementary benefits when selecting fastening strategies for CFRP structures.

## Figures and Tables

**Table 1:** Material properties [32].

Property	Carbon Fiber	Onyx
Tensile modulus (GPa)	60	2.4
Tensile strength (MPa)	800	40
Tensile strain (%)	1.5	25
Fiber diameter (μm)	150	-

**Table 2:** Summary of dimension and W/D, E/D.

D (mm)	W (mm)	E (mm)	W/D	E/D
5	20	30	4.0	6.0
6	20	30	3.3	5.0
8	20	30	2.5	3.8
10	20	30	2.0	3.0

D: Diameter of hole; W: Width of specimen; E: Distance of hole center to free edge.

**Table 3:** Laminate information of 3D printed bearing specimen.

Material	Printing Direction	**Total Layer No.**	**Total Layer Thickness (mm)**
Onyx	${\left[\pm 45{}^{\circ}\right]}_{2}$	4	0.5
Carbon fiber	${\left[0{}^{\circ}\right]}_{16}$	16	2.0
Onyx	${\left[\pm 45{}^{\circ}\right]}_{2}$	4	0.5
Onyx	${\left[\pm 45{}^{\circ}\right]}_{2}$	4	0.5
Carbon fiber	${\left[\pm 45{}^{\circ}\right]}_{8}$	16	2.0
Onyx	${\left[\pm 45{}^{\circ}\right]}_{2}$	4	0.5
Onyx	${\left[\pm 45{}^{\circ}\right]}_{2}$	4	0.5
Carbon fiber	${\left[90{}^{\circ}\right]}_{16}$	16	2.0
Onyx	${\left[\pm 45{}^{\circ}\right]}_{2}$	4	0.5

**Table 4:** Result of ultimate bearing stress, strain, stiffness, and fracture energy.

Type	Fiber Orientation	Diameter of Hole (mm)	Ultimate Bearing Stress (MPa)	Bearing Strain (-)	Structure Stiffness (N/mm)	Toughness (MJ/$m^{3}$)
Type 1	0°	5	148.85	1.7	1992.58	206.1
		6	140.81	1.05	1716.25	115.6
		8	129.96	0.65	1431.68	53.9
		10	110.21	0.31	1655.52	23.6
	±45°	5	157.51	1.69	2356.85	204.7
		6	138.45	1.31	1375.49	134.4
		8	88.12	0.67	1103.48	42.8
		10	76.90	0.27	1887.90	15.3
	90°	5	154.48	1.52	902.97	161.2
		6	125.04	0.88	1247.12	66.1
		8	102.65	0.59	785.37	33.4
		10	74.85	0.31	1469.37	15.4
Type 2	0°	5	154.78	3.92	2557.36	458.5
		6	173.23	3.22	6212.23	322.5
		8	128.10	0.75	4049.77	79.7
		10	83.24	0.05	20,618.48	3.8
	±45°	5	127.95	1.87	2274.71	216.2
		6	126.03	1.00	2634.86	96.9
		8	97.01	0.26	2478.66	10.1
		10	62.44	0.15	2683.59	5.9
	90°	5	148.91	1.19	2415.53	121.2
		6	132.16	0.54	2892.72	54.5
		8	109.99	0.43	3501.11	29.9
		10	82.21	0.09	8093.42	5.5

**Table 5:** Failure mode of 3D printed bearing specimen.

Case		Hole Diameter [W/D Ratio]			
**Type 1**		**5 mm [4.0]**	**6 mm [3.3]**	**8 mm [2.5]**	**10 mm [2.0]**
0°	No. 1	B	B	B	B
	No. 2	B	B	B	B
	No. 3	B	-	B	B
±45°	No. 1	B	B	B + T	B + T
	No. 2	B	B	B + T	B + T
	No. 3	B	B	B + T	B + T
90°	No. 1	B + N	B	B	B + N
	No. 2	B + N	B	B + C	B + N
	No. 3	B + N	B + C	B	B + N
**Type 2**		**5 mm [4.0]**	**6 mm [3.3]**	**8 mm [2.5]**	**10 mm [2.0]**
0°	No. 1	B	B	B	S
	No. 2	B	B	B	S
	No. 3	B	B	B	S
±45°	No. 1	B + T	B + T	B	T
	No. 2	B + T	B + T	B	T
	No. 3	B + T	-	B	-
90°	No. 1	B + N	B + N	B + N	C
	No. 2	B + N	B + C	B + N	C
	No. 3	B + C	B + N	B + N	C

B: Bearing failure mode; S: Shear-out failure mode; N: Net-tension failure mode; C: Cleavage failure mode; T: Tear-out failure mode.

## Data Availability

The original contributions presented in the study are included in the article; further inquiries can be directed to the corresponding author.
